# Outputs from the Healthy Communities programme

**DOI:** 10.1177/17579139241251740

**Published:** 2024-12-04

**Authors:** J Stansfield, J South

**Affiliations:** Leeds Beckett University, Calverley Building, Leeds, LS13HE, UK; Leeds Beckett University, Leeds, LS13HE

## Abstract

This paper brings together all the PHE outputs from a 10 year collaboration on Healthy Communities. This will help to retain the knowledge during organisational and government change.


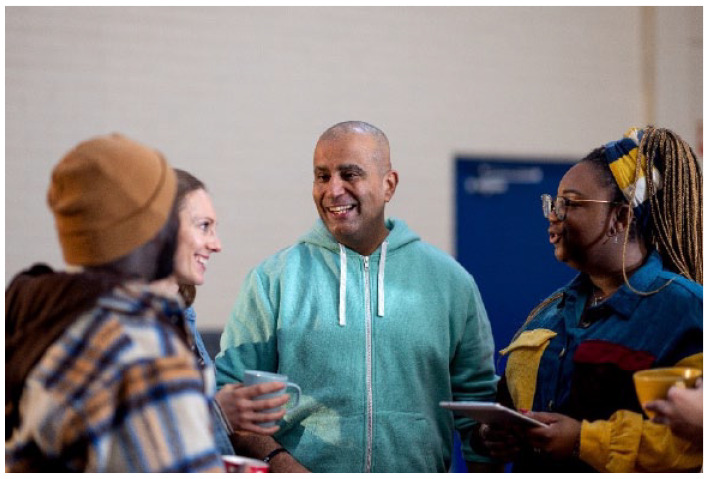


Community-centred approaches for health and wellbeing have gained increasing interest over recent years within public health, wider health and social care systems. Despite a long history of UK practice in community development for health, communities can still be on the receiving end of public health rather than in the driving seat.

Ten years ago, as Public Health England (PHE) was forming, there was a fragmented evidence base for community development and involvement in health. Authors formed a partnership to address this, which led to a 10-year collaboration between PHE and Leeds Beckett University. A small Healthy Communities programme, based in PHE with academic support, produced government guidance and supported its implementation into policy and practice through developing leadership and partnerships with national and regional bodies and local organisations working with communities. This sustained focus on knowledge translation of the best and most relevant evidence has created a significant body of knowledge and resources. It has been brought together for the first time into a descriptive bibliography available online https://eprints.leedsbeckett.ac.uk/id/eprint/10585/. This aims to support ongoing learning and mobilisation of evidence into policy and practice to meet future public health challenges.

Over this time, there have been other noteworthy contributions to advance evidence into practice and provide advocacy and system leadership for strengthening communities and coproduction approaches in public health, such as those included in this journal.^1^ Maintaining this agenda has been essential and challenging within the contexts of austerity, COVID-19, individualised health approaches, and social polarisation. Community factors – such as connectedness, cohesion, belonging, trust, and empowerment – are important influences on our mental and physical health, and potentially becoming increasingly significant as inequalities widen. Community-centred approaches can help address these factors and increase the control that communities have over the things that matter to their health, build trust and relationships between communities and with services, and lead to more effective and efficient mobilisation of public resources and community assets.^2^

We have learnt that the taxonomy of a ‘family of community-centred approaches’ has been useful to guide practice, especially in planning, commissioning, and scaling. The practice examples bring the ‘family’ to life and are a good source of learning on the mechanisms for success. While there is a range of approaches, different community health roles share many characteristics and require similar skills. The COVID-19 pandemic response included a growth in community health champion roles and a spotlight on the importance of communities and community-centred approaches to public health.^3^ The pandemic strengthened our learning on how systemic change requires more than scaling commissioned health inequalities’ interventions. Listening to communities, building trust, coproducing solutions, and measuring what matters to communities are important elements for change that address gaps.^4^

Tackling social disconnection is increasingly being seen as an urgent public health issue.^5^ It is not a discrete programme of work but a central pillar of action to reduce inequalities across all health outcomes and conditions, nor is it a euphemism for ‘targeting local populations’ or ‘engaging them’ in service design. It requires addressing those community-level factors, such as trust, as part of a social determinants of health approach and understanding the psychosocial pathways to health equity. It requires putting communities at the heart of everything we do in public health. We believe that a more community-centred public health system will help us meet the public health challenges of the next decade.

We continue to work with localities who are taking a whole system approach and embedding community-centred ways of working across all public health. Please get in touch if you would like to know more or share your learning.

Outputs from the Healthy Communities programme Leeds Beckett University and Public Health England: https://eprints.leedsbeckett.ac.uk/id/eprint/10585/.
